# Tangshenning Attenuates High Glucose-Induced Podocyte Injury via Restoring Autophagy Activity through Inhibiting mTORC1 Activation

**DOI:** 10.1155/2022/1610416

**Published:** 2022-06-28

**Authors:** Jiayi Xu, Xiaomeng Shan, Chunwei Chen, Yanbin Gao, Dawei Zou, Xiaolei Wang, Tao Wang, Yimin Shi

**Affiliations:** ^1^School of Traditional Chinese Medicine, Capital Medical University, Beijing, China; ^2^Beijing Key Lab of TCM Collateral Disease Theory Research, Beijing, China

## Abstract

Diabetic nephropathy (DN) is a microvascular complication of diabetes mellitus (DM) and the most common cause of death in diabetic patients. DN progression is associated with podocyte damage due to reduced autophagy caused by mTORC1 activation. Tangshenning (TSN) has been shown to reduce proteinuria, protect renal function, and reduce podocyte damage. Still, the effect of TSN on the autophagic activity of podocytes remains unclear. Herein, *in vitro* experiments using a high glucose-induced podocyte injury model were performed. Results showed that TSN treatment enhanced the weakened nephrin expression and autophagic activity of podocytes and inhibited the mTORC1 pathway (p-mTOR, mTOR, p-p70S6K, p70S6K, ULK1, and 4EBP1) under high glucose conditions. Furthermore, the mTORC1 activator (siRNA-TSC2) partially inhibited the above beneficial effects of TSN, suggesting that mTORC1 was the target of TSN to regulate autophagy. In summary, TSN reduces podocyte damage induced by high glucose via inhibiting mTORC1 pathway and downstream targets and restoring podocyte autophagy.

## 1. Introduction

Diabetic nephropathy (DN), a severe microvascular complication of diabetes, is the main cause of chronic kidney disease (CKD) and end-stage renal disease (ESRD) [1]. The pathogenesis of DN is multiple and complex, but the exact mechanism has not been fully elucidated. In recent years, podocytes have attracted much attention due to their nonrenewability [2]. Podocytes are the last barrier of the glomerular filtration membrane, and podocyte dysfunction can cause proteinuria and glomerular sclerosis, exacerbating the progression of DN [3]. Experimental research found a markedly decreased expression of autophagy-related proteins such as Beclin-1, Atg12-Atg5, and LC3-II in a diabetic mouse model and in a high glucose-induced podocyte injury model [4]. Podocyte damage caused by weak autophagic activity is an essential mechanism underlying DN progression [5].

Autophagy is a highly conserved and lysosome-dependent bulk degradation process. Autophagy is the “self-eating” process of degrading damaged proteins and organelles, as well as recycling intracellular energy to maintain cellular homeostasis under stress conditions. Podocytes depend on high autophagic activity to maintain their physiological functions [6]. The mammalian target of rapamycin (mTOR) is an evolutionarily highly conserved intracellular serine/threonine kinase family member. It mainly forms the catalytic subunits of two protein complexes, including mTOR complex 1 (mTOR complex1, mTORC1) and mTOR complex 2 (mTOR complex2, mTORC2). mTORC1 activation inhibits autophagy, and it is considered to be a molecular marker of diabetic nephropathy [7, 8], as the decrease in podocyte autophagic activity caused by mTORC1 activation plays a vital role in the progression of this disease [9]. Inhibiting mTORC1 in podocytes thus could be the key to prevent and treat DN.

Tangshenning formula (TSN), a Chinese medicine, mainly includes Astragali Radix, Rhei Radix Et Rhizoma, Chuanxiong Rhizoma, and Rosae Laevigatae Fructus. TSN has been applied clinically for many years to reduce proteinuria and improve renal function in patients with DN, and its therapeutic effects have been confirmed in clinical trials [10]. TSN has also been confirmed to reduce podocyte damage in DN rats and high glucose-cultured podocytes [11, 12]. Experimental studies have shown that the main bioactive components of TSN, astragaloside IV, calycosin, emodin, ferulic acid and quercetin have beneficial effects on diabetic nephropathy by reducing proteinuria, protecting renal function, and inhibiting renal fibrosis [13–18]. Besides, studies have shown that astragaloside IV, emodin, rhein, ferulic acid, ligustilide, and quercetin can reduce podocyte damage in diabetic nephropathy [18–23]. More importantly, astragaloside IV (the main component of Astragali Radix) and emodin (the main component of Rhei Radix Et Rhizoma) ameliorate podocyte damage in diabetic nephropathy by enhancing podocyte autophagy [13, 16]. Ferulic acid, the main component of Chuanxiong Rhizoma, reduces hyperglycemia-induced kidney damage through its anti-inflammatory and antioxidant activities and its role in enhancing autophagy [24]. However, the mechanism by which TSN works has not been established. This study aims to determine whether the protective effect of TSN on podocytes is related to inhibiting the mTORC1 pathway and restoring podocyte autophagic activity.

## 2. Materials and Methods

### 2.1. Components and Quality Control of TSN

**Figure 1:**
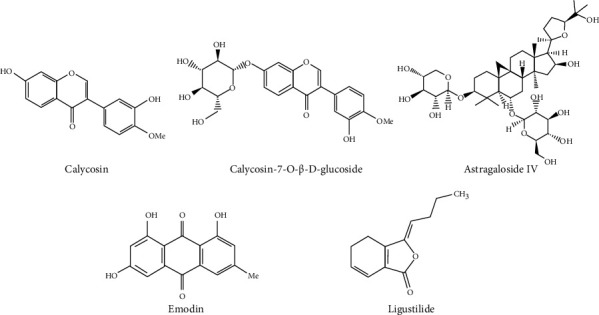
The chemical structure of five main compounds.

**Figure 2:**
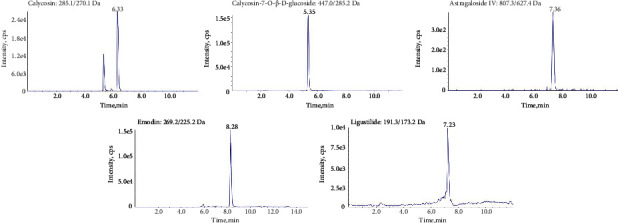
Typical UPLC-ESI-MS/MS chromatograms of five bioactive components in TSN.

TSN came from a Chinese medicine factory (Beijing Tcmages Pharmaceutical Co. Ltd., Beijing, China). TSN is composed of Astragali Radix, Rhei Radix Et Rhizoma, Chuanxiong Rhizoma, and Rosae Laevigatae Fructus. Each herbal plant contains several main bioactive components. Astragali Radix mainly contains astragaloside IV, astragaloside III, calycosin, and calycosin-7-O-*β*-D-glucoside. Rhei Radix Et Rhizoma mainly contains emodin, rhein, aloe-emodin, and physcion. Chuanxiong Rhizoma mainly contains ligustilide and ferulic acid. Rosae Laevigatae Fructus mainly contains quercetin and kaempferol. The development of high-performance liquid chromatography-tandem mass spectrometry (HPLC-MS/MS) has been used to study traditional Chinese medicine compound [25]. In the present study, ultraperformance liquid chromatography with electrospray ionization coupled with tandem mass spectrometry (UPLC-ESI-MS/MS) method was used to qualitatively and quantitatively analyze these five bioactive components of TSN. The chemical structures of the five major compounds are shown in Figure 1. The contents of the bioactive components were 104.9, 91.9, 62.9, 40.7, and 22.8 *μ*g/g for calycosin, calycosin-7-O-*β*-D-glucoside, astragaloside IV, emodin, and ligustilide, respectively. Typical LC-MS/MS chromatograms of five bioactive components of TSN are shown in Figure 2.

### 2.2. Preparation of Rat Serum-Containing Drug

All experimental studies were approved by the Ethics Review Committee for Animal Experimentation of Capital Medical University (Ethical Approval Number AEEI-2017-039). Male SPF grade Sprague-Dawley (SD) rats (license number: SCXK (Jing) 2016-0006) were obtained from Beijing Vital River Laboratory Animal Technology Company (Beijing, China). Rats were randomly divided into the normal group and the TSN group. Rats of the TSN group were administered with TSN intragastrically (20 g/kg/day) once a day for 7 days. Rats in the normal group were gavaged with an equal volume of normal saline as control. One hour after the last administration, serum was collected and stored at -80°C. The rat serum was used in subsequent tests.

### 2.3. Cell Culture

The conditional immortalized mouse podocyte cell line (BNCC337685) was purchased from BeNa Culture Collection (Beijing, China) [19]. The podocytes were cultured in DMEM low glucose medium (Gibco, USA) containing 10% fetal bovine serum (FBS, ExCell Bio, China) and recombinant interferon-gamma at 33°C, 5% CO2. Podocyte differentiation was induced without IFN-*γ* at 37°C. The differentiated podocytes were cultured in DMEM medium without FBS for 24 hours before being exposed to various experimental conditions and then divided into the following groups: (1) normal group (NG), 5.5 mM glucose + normal rat serum; (2) mannitol group (MG), 5.5 mM glucose + 24.5 mM D-mannitol + normal rat serum; (3) model group (HG), 30 mM glucose + normal rat serum; (4) low-dose TSN group (LT), 30 mM glucose +1/25 rat serum with TSN; (5) medium-dose TSN group (MT), 30 mM glucose +1/5 rat serum with TSN; and (6) high-dose TSN group (HT), 30 mM glucose +1 rat serum with TSN.

### 2.4. Cell Viability

Cell viability was performed to detect the effect of TSN and to determine the optimal TSN treatment concentration. Cell viability of podocytes was evaluated using a CCK-8 assay kit (Beyotime, Shanghai, China). Different concentrations of rat serum with TSN (1/50, 1/25, 1/10, 1/5, 1/2, and 1) were added and incubated for 24 h, 48 h, and 72 h. Finally, CCK8 solution was added to incubate for 2 h, and the cell survival rate was determined by measuring the OD value of 450 nm with a microplate reader (SpectraMax iD3, SpectraMax, Austria). The cell viability was quantified with the following formula: (OD EXP–OD blank)/(OD NG–OD blank) × 100% [26].

### 2.5. Cell Transfection with TSC2 siRNA

Tuberous sclerosis complex 2 (TSC2) is an upstream negative regulator of mTORC1, and knocking down TSC2 can activate the mTORC1 pathway. TSC2 small interfering RNA (TSC2 siRNA) was purchased from Ribobio (Guangzhou, China). The three target sequences of mouse TSC2 siRNA (5′-3′) were as follows: TSC2 siRNA1: CCATTAAGGGCCAGTTCAA; TSC2 siRNA2: CAAGACTCATCAAGAAGTA; TSC2 siRNA3: GCTGTTACCTTGACGAATA. According to the manufacturer's instructions, podocytes were transfected with TSC2 siRNA, positive siRNA (Si PC), or negative control siRNA (Si NC) for 24 h. The effective transfection sequence was further analyzed and selected by Western blotting. After testing and comparing the three siRNAs, TSC2 siRNA3 was selected for subsequent experiments.

TSC2-siRNA3 was used to study the mechanism by which TSN regulates autophagy and reduces podocyte damage. Cells were treated in transfected or nontransfected conditions, and grouped as follows: (1) NG; (2) MG; (3) HG; (4) 30 mM glucose and TSN (30 mM glucose +1 rat serum with TSN, HG+TSN), (5) 30 mM glucose negative control siRNA transfected (30 mM glucose + NC siRNA, HG+NC), (6) 30 mM glucose-negative control siRNA transfected and TSN (30 mM glucose + NC siRNA +1 rat serum with TSN, HG+NC+TSN), and (7) 30 mM glucose TSC2 siRNA transfected and TSN (30 mM glucose + TSC2 siRNA +1 rat serum with TSN, HG+TSC2+TSN).

### 2.6. Electron Microscopy of Autophagosomes

The samples were prefixed in 2.5% glutaraldehyde for 2 hours, rinsed three times with PBS, and postfixed in 1% osmium acid at 4°C for 2 hours. After the resin and acetone were soaked, embedded, and polymerized, the embedded block was semithinly positioned and ultrathinly sectioned. Finally, after the counterstaining of uranyl acetate and lead citrate, the ultrastructure of the podocytes was observed and photographed under a transmission electron microscope (JEM-2100, JEOL, Tokyo, Japan).

### 2.7. Western Blot Analysis

Podocytes were lysed in RIPA buffer on ice for 10 min to extract total proteins. Proteins were measured with a bicinchoninic acid (BCA) protein assay kit. The loaded proteins were separated by electrophoresis, transferred to a PVDF membrane, and blocked with TBST containing 5% skim milk for 1-2 h, and the primary antibody was incubated overnight at 4°C. The primary antibody concentrations were as follows: mouse anti-GAPDH (Proteintech, 1 : 5000), rabbit anti-LC3A/B (CST, 1 : 1000), mouse anti-P62 (Abcam, 1 : 10000), rabbit anti-nephrin (Novus, 1 : 1000), rabbit anti-mTOR (CST, 1 : 1000), rabbit anti-p-mTOR (CST, 1 : 1000), rabbit anti-ULK1 (CST, 1 : 1000), rabbit anti-TSC2 (CST, 1 : 1000), rabbit anti-4EBP1 (CST, 1 : 1000), rabbit anti-p70S6K (CST, 1 : 1000), and rabbit anti-p-p70S6K (CST, 1 : 1000). The next day, the membranes were incubated with the corresponding secondary antibody and finally exposed using a gel imaging system (FUSION FX6 XT, Vilber, France).

### 2.8. Immunofluorescence

The podocyte slides were inoculated in a 12-well plate and given different treatments for 24 hours and then fixed in 4% paraformaldehyde solution for 15 minutes. The slides were incubated with 0.5% Triton X-100 for 20 min and then blocked with 10% goat serum for 30 minutes. Next, the slides were incubated with a nephrin antibody (Novus, 1 : 100) and LC3 II primary antibody (CST, 1 : 100) overnight at 4°C. The next day, the fluorescent secondary antibody was incubated at 37°C for 1 hour. Finally, the images were observed and collected under a fluorescence microscope (DM60008/TCS SP8 STED, Leica, Wetzlar, Germany).

### 2.9. Phalloidin Staining

The podocyte slides were inoculated and given different treatments for 24 hours and then fixed with 4% paraformaldehyde solution for 20 minutes. Slides were incubated for 10 min in a PBS solution containing 0.1% TritonX-100. Then, 100 *μ*g/ml rhodamine phalloidin (AAT Bioquest, Sunnyvale, CA, USA) was added and incubated at room temperature for 1 hour. Finally, cells were photographed under a laser confocal microscope (TCS SP8 STED, Leica, Wetzlar, Germany).

### 2.10. Statistical Analysis

Statistical analysis was performed using Prism software version 7 (GraphPad, San Diego, CA). Differences between groups were analyzed using t-test and one-way ANOVA. The data were expressed as mean ± standard deviation ($\bar{\text{X}}\pm \text{SD}$). *P* < 0.05 was considered statistically significant.

## 3. Results

### 3.1. TSN Increased Podocyte Cell Viability after High Glucose Induction *In Vitro*

**Figure 3:**
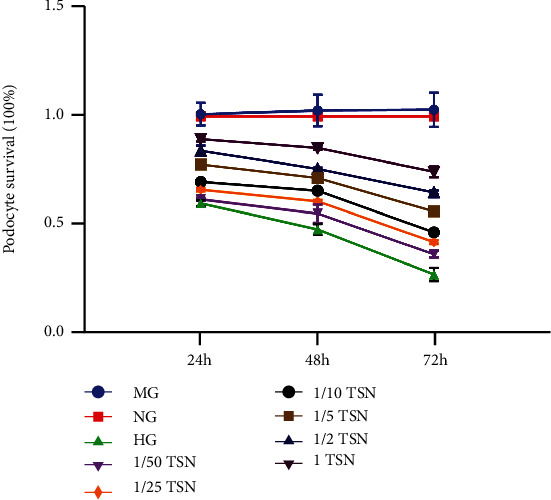
Effect of TSN on the viability of high glucose-induced podocytes. CCK-8 assay results showed that TSN increased the viability of the podocyte injury model induced by high glucose. The podocytes were cultured for 24 h, 48 h, and 72 h with 30 mM high glucose and different concentrations of TSN ((1/50, 1/25, 1/10, 1/5, 1/2, and 1). Cell viability is expressed as mean ± SD (*n* = 3).

The effect of TSN on high glucose-induced podocyte injury *in vitro* was observed in rat serum with TSN at six concentrations (1/50, 1/25, 1/10, 1/5, 1/2, and 1) and at three time points (24 h, 48 h, and 72 h) (Figure 3). CCK-8 results showed that mannitol had no significant effect on podocyte viability (*P* > 0.05), which could dismiss the effect of osmotic pressure on podocyte damage caused by high glucose. At three different time points, high glucose significantly reduced the viability of podocytes (*P* < 0.05), while TSN could reverse the decrease in podocyte viability induced by high glucose (*P* < 0.05). However, the effect of 1/50 TSN on reversing the decrease in podocyte viability was pronounced at 72 h (*P* < 0.05), but not at 24 h and 48 h (*P* > 0.05). According to the above experimental results, the cell viability dropped to about 60% after 24 h high glucose incubation. Compared with the low cell viability of 48 h and 72 h, the cell viability of 24 h is more suitable as a time point to study the mechanism of TSN. TSN treatment for 24 h showed a significant dose-dependent effect of increasing cell viability, and 1 TSN had the best effect (*P* < 0.05). Therefore, 1/25, 1/5, and 1 TSN were selected as the low, medium, and high concentrations for subsequent TSN treatment, and 24 h was set as the final treatment time.

### 3.2. TSN Attenuated Podocyte Cytoskeletal Injury and Enhanced Nephrin Protein Expression after High Glucose Induction *In Vitro*

**Figure 4:**
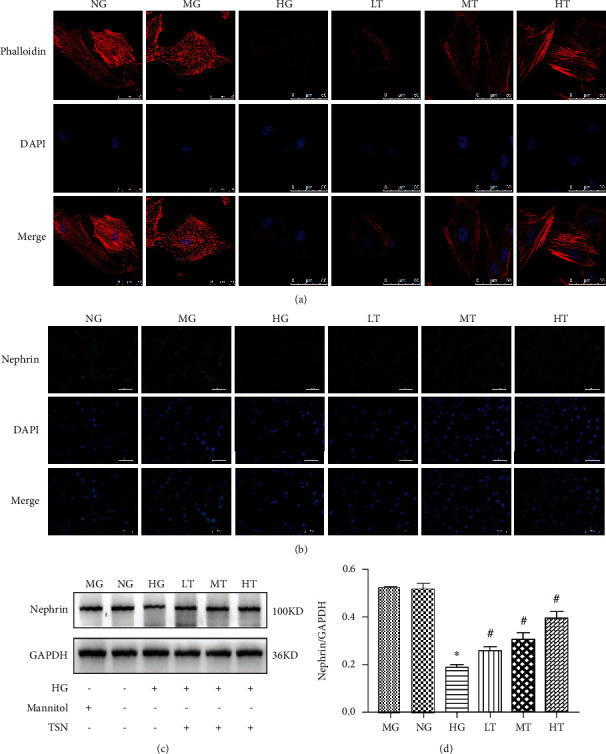
Effects of TSN on nephrin expression and podocyte cytoskeleton in high glucose-induced podocytes. (a) Staining of podocytes cytoskeleton with phalloidin, original magnification ×630, scale 50 *μ*m, *n* = 3. (b) Representative immunofluorescence photographs of nephrin in each group, magnification ×400, scale 50 *μ*m, *n* = 3. (c–d) Representative Western blot band and quantification of nephrin protein expression in each group, data were shown as the mean ± SD, *n* = 3; ∗*P* < 0.05 versus the NG group, #*P* < 0.05 versus the HG group.

To observe the effect of TSN on the skeletal structure of podocytes caused by high glucose, phalloidin was used to stain the podocyte actin cytoskeleton. As shown in Figure 4(a), the normal cytoskeleton is a network structure composed of actin fiber microfilaments, which were linearly and parallelly arranged along the long axis of the cell, neat and clear. The actin filaments of podocytes induced by high glucose showed collapse or breakage, and the skeletal structure shrank. The cytoskeleton structure was partially restored by the treatment of TSN.

Decreased nephrin expression is a hallmark of podocyte damage. To evaluate the effect of TSN on podocyte damage, immunofluorescence staining and Western blot were applied to detect nephrin protein expression. Nephrin protein is expressed in the cytoplasm and distributed in granular or linear form. The fluorescence intensity of the HG group was significantly lower than that of the NG group, and it was significantly increased by the TSN treatment (Figure 4(b)). Western blot results showed that high glucose could reduce nephrin expression (*P* < 0.05), consistent with the immunofluorescence results. Furthermore, an increase in nephrin expression was associated with TSN treatment, and the HT group had the best effect (*P* < 0.05) (Figures 4(c) and 4(d)). Therefore, the HT group was set as the final concentration to study the mechanism of autophagy regulation. In short, TSN could improve podocyte structure injury and increase nephrin expression.

### 3.3. TSN Attenuated High Glucose-Induced Reduction of Podocyte Autophagy

**Figure 5:**
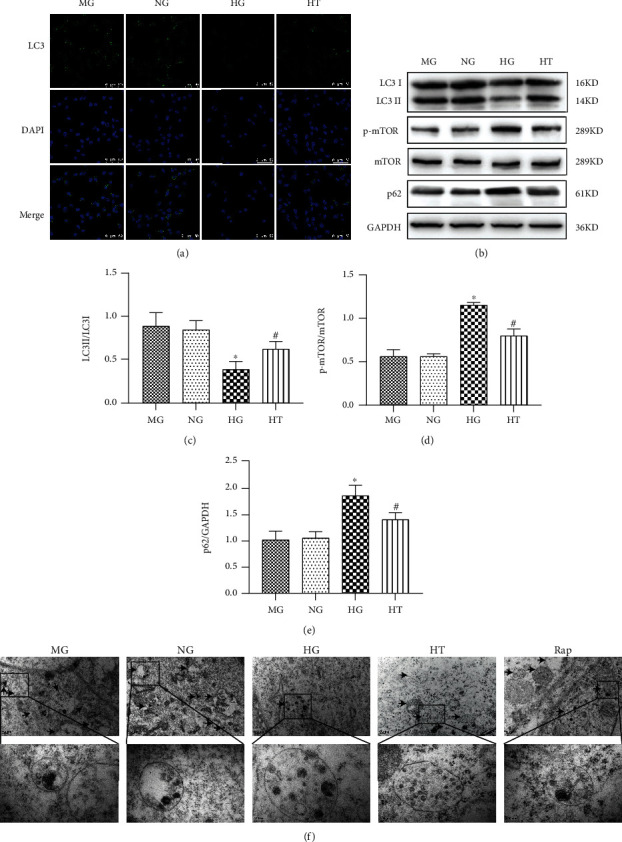
Effect of TSN on the autophagic activity of high glucose-induced podocytes. (a) Representative immunofluorescence photographs (magnification ×630) of LC3II in each group (*n* = 3). Scale bar =50 *μ*m. (b–e) Representative Western blot bands and quantification of LC3II/I, p-mTOR, mTOR, and P62 protein expression. Data were shown as mean ± SD, n = 3; ∗*P* < 0.05 versus the NG group, #*P* < 0.05 versus the HG group. (f) Electron microscope observation showed that the number of autophagosomes decreased under high glucose conditions, and TSN treatment could improve it. The scales and magnifications are 0.2 *μ*m and ×6000 for the upper picture and 100 nm and ×20000 for the lower picture.

The activation of the mTOR pathway was negatively correlated with autophagic activity. mTOR, p-mTOR, LC3II, and P62 were detected by Western blot or immunofluorescence. p-mTOR/mTOR expression was increased in HG-cultured podocytes. Surprisingly, the TSN treatment significantly decreased the p-mTOR/mTOR expression (*P* < 0.05). Moreover, the data showed that the TSN treatment reversed the decrease in the LC3II/LC3I ratio and the increase in P62 expression caused by hyperglycemia (*P* < 0.05) (Figures 5(a)–5(e)). In short, TSN treatment could inhibit mTOR pathway activation and attenuate the reduction of podocyte autophagic activity.

Electron microscopy observation of autophagosomes is a widely used method to study the autophagic activity of cells. As shown in Figure 5(f), the podocyte shows a clear autophagosome structure, confirming the occurrence of autophagy. Besides, it was observed that the number of autophagosomes in the HG group decreased, and TSN showed a similar effect in increasing the number of autophagosomes as rapamycin.

### 3.4. Selection of the Most Effective TSC2-siRNA Sequence Targeting TSC2

**Figure 6:**
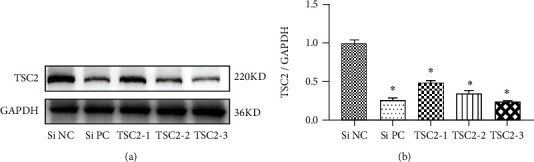
Detection of siRNA TSC2 knockdown efficiency in podocytes. (a–b) Representative Western blot bands and quantification of TSC2 protein expression. Podocytes were transfected with TSC2 siRNA, positive siRNA (Si PC), or negative control siRNA (Si NC). Data were shown as the mean ± SD, *n* = 3; ∗*P* < 0.05 versus the Si NC group.

To evaluate the knockdown efficiency of siRNA-TSC2 at the level of TSC2, high glucose-cultured podocytes were transfected with three different siRNA-TSC2 sequences. After TSC2 siRNA treatment for 24 h, Western blot analysis showed that transfection of TSC2 siRNA-1, TSC2 siRNA-2, and TSC2 siRNA-3 significantly reduced the expression of TSC2 in podocytes cultured with high glucose (*P* < 0.05). Compared with the Si NC group, the knockdown efficiency of TSC2 siRNA1, TSC2 siRNA2, and TSC2 siRNA3 was close to 51.2%, 65.2%, and 75.6% (Figure 6). Based on the above results, TSC2 siRNA3 was selected for transfection in subsequent experiments.

### 3.5. TSN Protected Podocytes from Impairment of Autophagy Induced by High Glucose via the mTORC1 Pathway

**Figure 7:**
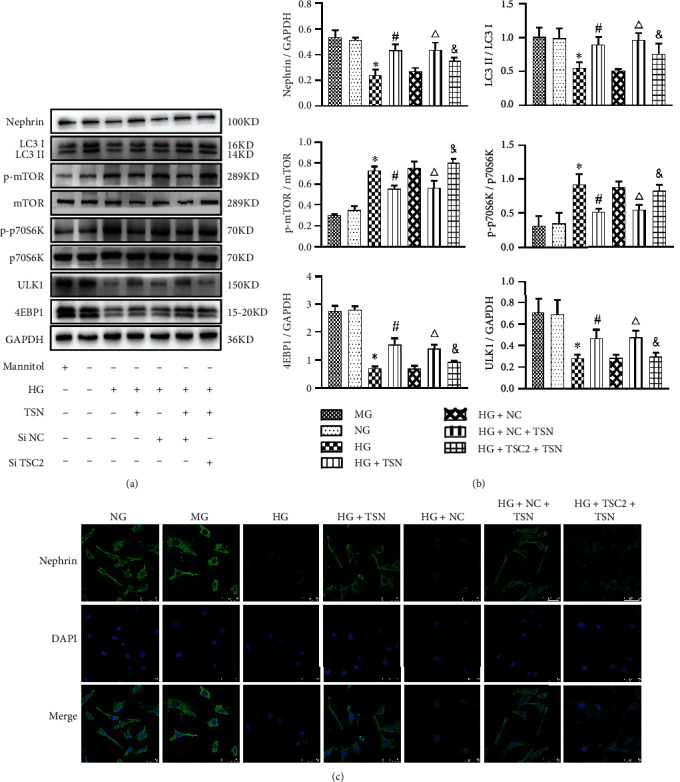
Effect of TSN on the mTORC1 activation, LC3II, and nephrin in high glucose-induced podocytes. (a–b) Representative Western blot bands and quantification of nephrin, LC3II/I, mTOR, p-mTOR, p-p70S6K, p70S6K, ULK1, and 4EBP1 protein expression. Data were shown as the mean ± SD, *n* = 3; ∗*P* < 0.05 versus the NG group, #*P* < 0.05 versus the HG group, △*P* < 0.05 versus the HG + NC group, ^&^*P* < 0.05 versus the HG + NC+ TSN group. (c) Representative immunofluorescence photographs of nephrin in each group, magnification ×630, scale 50 *μ*m, *n* = 3.

We further aimed to determine whether TSN attenuates the decline of the podocyte autophagic activity by regulating the mTORC1 pathway and downstream targets. After high glucose exposure, cells were treated with TSC2 siRNA and negative siRNA and then with TSN for 24 h. p70S6K, 4EBP1, and ULK1 are key downstream effectors of mTORC1 activation; mTORC1 positively regulates p70S6K and negatively regulates ULK1 and 4EBP1. Compared with the NG group, p-mTOR and p-p70S6K expression increased (*P* < 0.05), and ULK1, 4EBP1, LC3II, and nephrin expression decreased in the HG group (*P* < 0.05), suggesting that the mTORC1 pathway was activated by high glucose. TSN treatment (HG + TSN) could reverse the activation of the mTORC1 pathway, which was accompanied by the increase of LC3II and nephrin expression (*P* < 0.05). Meanwhile, we used TSN and siRNA-TSC2 to incubate high glucose-induced podocytes (HG + TSC2 + TSN group) and found that the effect of TSN on enhancing LC3II and nephrin protein expression was partially inhibited by mTORC1 pathway activation (*P* < 0.05) (Figures 7(a)–7(c)). The results suggested that the mTORC1 activator (siRNA-TSC2) could inhibit the effect of TSN on enhancing autophagy and reducing podocyte damage. Based on the above experimental results, TSN could restore podocyte autophagic activity and reduce podocyte damage by inhibiting the mTORC1 pathway and downstream targets in the high glucose-induced podocyte injury model.

## 4. Discussion

This study confirmed the effectiveness of TSN in reducing podocyte damage induced by high glucose and found that the mechanism is partly associated with inhibiting the mTORC1 pathway and restoring podocyte autophagic activity. DN is the leading cause of death in diabetic patients; however, the multiple and complex pathogenesis of DN has not been fully elucidated, and its prevention and treatment remain an arduous task [27, 28]. Decreased podocyte autophagic activity is considered a key factor in promoting podocyte damage, proteinuria, and deterioration of renal function [29]. Therefore, podocyte autophagic activity has become a research hotspot in the field of diabetic nephropathy recently. The mTORC1 pathway is an important pathway that regulates the autophagic activity of podocytes. Rapamycin, a mTORC1 inhibitor, can promote podocyte autophagy to improve diabetic kidney damage [30]. However, its effect has not achieved satisfactory results in clinical trials, which dismissed the possibility of widespread clinical application. Therefore, effective drugs for the treatment of DN are urgently needed. TSN has been clinically effective in treating DN for many years, but the mechanism of how it reduces podocyte damage has not been fully elucidated.

In this study, *in vitro* experiments confirmed that TSN can reduce podocyte damage caused by high glucose. We mainly studied the effects of TSN on cell viability and evaluated podocyte damage using nephrin and cytoskeleton integrity as markers. TSN improves cell viability of podocytes under high glucose condition. It has been reported that the viability of podocytes is related to the down-regulation of extracellular high glucose-dependent autophagy [31]. The podocyte split membrane comprises various proteins such as nephrin, which connect adjacent foot processes to form a zipper-like structure, forming a selective filtration barrier for proteins and other macromolecular substances. The nephrin expression of podocytes is significantly reduced in diabetic nephropathy. In this study, WB and immunofluorescence were used to confirm the protective effect of TSN on the decrease in nephrin expression under high glucose conditions, consistent with previous *in vivo* experiments [11]. In addition, glomerular podocyte skeletal actin is essential for maintaining the stability of the cytoskeleton structure and normal glomerular filtration function [32]. Phalloidin staining results confirmed that TSN improved the unclear cytoskeletal structure and disordered arrangement.

This study confirmed that TSN could enhance the autophagic activity of podocytes cultured with high glucose. Podocytes are differentiated, mature cells with limited proliferation ability, so the self-repair mechanism is important in maintaining the stability of the internal environment. Autophagy is also a housekeeping mechanism by which cells maintain a steady state and avoid apoptosis and necrosis. Hence, decreased podocyte autophagic activity can lead to podocyte damage [5, 33]. The microtubule-associated protein 1 light chain 3 II (LC3 II) and P62 are considered the markers of autophagic activity. LC3II marks autophagosome formation, and p62 marks autophagolysosome degradation [34]. In the podocytes induced by high glucose in this experiment, the expression of P-mTOR and P62 increased, and the expression of LC3II decreased, which is consistent with another study [35]. It is reported that kidney biopsy specimens of patients with DN showed P62 protein accumulation in the glomerulus, which is related to proteinuria [36]. Aggregation of P62, a substrate for autophagy degradation, indicates insufficient autophagy activity of podocytes. More importantly, TSN reversed the expression trend of P-mTOR, LC3II, and P62 in podocytes exposed to high glucose and partially restored the autophagy activity of podocytes. It is reported that astragaloside IV, emodin, and ferulic acid can regulate the abnormal expression of LC3II and P62 in diabetic animals or high glucose-incubated podocytes [13, 16, 24]. These studies support the effect of TSN on enhancing autophagy from the perspective of active ingredients. Autophagy activity is a dynamic process. A study conducting observations on the autophagy activity of podocytes for different periods of time confirmed the short-term induction and long-term inhibition of podocyte autophagy by high glucose [13]. However, the autophagic activity of podocytes at different time points was not dynamically observed in our study, and further research is needed.

It is reported that mTORC1 is highly activated in the podocytes of humans and animals with DN, and the inactivation of mTORC1 improves the podocyte damage [37, 38]. Therefore, we focused on the mTORC1 pathway and downstream targets in our research. In conditions of hyperglycemia and oxidative stress, activated mTORC1 pathway can regulate podocyte mRNA translation, ribosomal biosynthesis, and protein translation through two key downstream effectors p70 S6 kinase (p70S6K) and 4E-binding protein 1 (4EBP1), thereby inhibiting autophagy [39, 40]. To further identify the target of TSN, we used the siRNA method. TSC2 is the negative regulator at the upstream of mTORC1, and siRNA TSC2 can activate the mTORC1 pathway [7, 41]. Our study confirmed that TSN can partially reverse the reduction of podocyte autophagy activity by inhibiting the activation of mTOR and P70S6K and the inactivation of 4EBP1, a finding similar to another research on the effect of rapamycin [42]. Astragaloside IV, the main active component of TSN, can also inhibit the phosphorylation expression of mTOR and p70S6K in podocytes treated with HG and enhance autophagy activity to reduce podocyte damage [13]. UNC51-like kinase (ULK1), which inhibits autophagy, is a substrate of mTORC1 [37]. TSN can reverse the down-regulation of ULK1 expression caused by mTORC1 activation. In addition, after siRNA TSC2 pretreatment, the effect of TSN in restoring the weakened autophagic activity and attenuating podocyte injury was partially inhibited, which proved that mTORC1 was the target of TSN to restore autophagic activity. Notably, the effect of TSN in reducing podocyte damage was not entirely blocked by activation of mTORC1, indicating that TSN could reduce podocyte damage in other ways. Therefore, other mechanisms for TSN to reduce podocyte damage need to be further studied.

## 5. Conclusions

This study confirmed that TSN could reduce podocyte damage induced by high glucose, and its mechanism is partly related to the inhibition of the mTORC1 pathway and restoration of podocyte autophagic activity. We further confirmed that the reduction of autophagy caused by mTORC1 activation plays an important role in podocyte injury.

## Data Availability

Data used to support the findings of this study are available from the corresponding author upon request.
